# FOXM1 promotes the progression of prostate cancer by regulating PSA gene transcription

**DOI:** 10.18632/oncotarget.15224

**Published:** 2017-02-09

**Authors:** Youhong Liu, Yijun Liu, Bowen Yuan, Linglong Yin, Yuchong Peng, Xiaohui Yu, Weibing Zhou, Zhicheng Gong, Jianye Liu, Leye He, Xiong Li

**Affiliations:** ^1^ Center for Molecular Medicine, Xiangya Hospital, Central South University, China; ^2^ Hunan Key Laboratory of Molecular Radiation Oncology, Xiangya Hospital, Central South University, China; ^3^ Department of Oncology, Xiangya Hospital, Central South University, China; ^4^ Department of Pharmacy, Xiangya Hospital, Central South University, China; ^5^ Department of Urology, The Third Xiangya Hospital, Central South University, China; ^6^ Research Institute of Prostate Diseases, Central South University, China

**Keywords:** PSA, FOXM1, gene transcription, androgen receptor, prostate cancer

## Abstract

Androgen/AR is the primary contributor to prostate cancer (PCa) progression by regulating Prostate Specific Antigen (PSA) gene transcription. The disease inevitably evolves to androgen-independent (AI) status. Other mechanisms by which PSA is regulated and develops to AI have not yet been fully determined. FOXM1 is a cell proliferation-specific transcription factor highly expressed in PCa cells compared to non-malignant prostate epithelial cells, suggesting that the aberrant overexpression of FOXM1 contributes to PCa development. In addition to regulating AR gene transcription and cell cycle-regulatory genes, FOXM1 selectively regulates the gene transcription of KLK2 and PSA, typical androgen responsive genes. Screening the potential FOXM1-binding sites by ChIP-PCR, we found that FOXM1 directly binds to the FHK binding motifs in the PSA promoter/enhancer regions. AI C4-2 cells have more FOXM1 binding sites than androgen dependent LNCaP cells. The depletion of FOXM1 by small molecular inhibitors significantly improves the suppression of PSA gene transcription by the anti-AR agent Cadosax. This is the first report showing that FOXM1 promotes PCa progression by regulating PSA gene transcription, particularly in AI PCa cells. The combination of anti-AR agents and FOXM1 inhibitors has the potential to greatly improve therapy for late-stage PCa patients by suppressing PSA levels.

## INTRODUCTION

Prostate cancer (PCa) is the most commonly diagnosed malignancy and the second leading cause of cancer death in males in the United States [1]. An estimated 180,890 new cases will be diagnosed and 26,120 men will die of the disease in 2016 [2]. PSA, a widely used marker for PCa early screening and for assessing patient therapeutic response, is not sufficient to predict tumor aggressiveness, resulting in over-diagnosis and over-treatment [3].

Androgen/androgen receptor (AR), the primary regulator of PSA, is essential for PCa initiation and progression. Androgen deprivation therapies (ADT) using luteinizing hormone-releasing hormone agonists or antagonists, such as flutamide or casodex, decrease PSA levels and slow disease progression [4, 5] but tumor regression is temporary, and PCa evolves to androgen-independent (AI) status. PCa is typically refractory to current treatments at this stage and the disease becomes lethal.

AR mediates androgen-induced signaling by binding to androgen responsive elements (AREs, GGTACAnnnTGTT/CCT) in the enhancers and promoters of AR-regulatory genes. Forming a productive AR transcriptional complex requires the functional and structural interaction of AR and its coregulators. The AR coregulators regulate gene transcription by directly binding to the *cis*-regulatory regions of target genes for DNA occupancy, chromatin remodeling, and/or recruitment of general transcription factors associated with RNA polymerase II, or by assuring the competency of the AR to enhance the gene expression directly [6]. Recent evidence indicates that the program of gene expression regulated by AR in the absence of hormone is distinct from the androgen-regulated program. In AD PCa cells, AR promotes cell proliferation by regulating the cell cycle G1/S transition in the presence of androgen, while in AI PCa, AR promotes AI disease progression through a variety of potential mechanisms including AR amplification or mutation, increased androgen sensitivity, local androgen production and growth factor activation [4, 7].

The forkhead box (FOX) superfamily of evolutionarily conserved transcription factors controls a wide spectrum of biological processes. Emerging evidence suggests that deregulation of FOX transcription factors is crucial in tumorigenesis and cancer progression [8]. Several FOX family members such as FOXO1, FOXA1 and FOXA2 and FOXH1 [9] structurally and functionally interact with AR as coregulators in PCa [10]. Recent evidence also suggests that FOXM1 has a role in cancer invasion and angiogenesis [11]. Elevated expression of FOXM1 has been especially observed in hormone-refractory and metastatic PCa tumor specimens [12]. Ectopic FOXM1 overexpression accelerates the development, proliferation, and growth of PCa in mouse models [13]. Thus FOXM1 is considered a promising target for therapeutic intervention in cancer [8], and its inhibitors, siomycin A and cell-penetrating ARF [26–44] peptide, induce cancer cell apoptosis [14, 15]. Our previous studies demonstrated that FOXM1 and AR co-activate CDC6 gene transcription and DNA replication in PCa cells through several pathways. FOXM1 regulates AR gene transcription, and FOXM1 protein interacts with AR protein, enhancing FOXM1 protein binding to CDC6 promoter [16].

This study focused on how FOXM1 and AR mutually regulate and co-activate PSA gene transcription. We first compared FOXM1 expression in malignant and non-malignant prostate epithelial cells. Then we tested FOXM1 transcription regulation of cell cycle-regulatory genes and androgen/AR regulatory genes. Screening potential FOXM1-binding sites by ChIP-PCR, we validated the binding of FOXM1 to PSA enhancer/promoter. Interestingly, we found many more FOXM1 binding sites in androgen-independent C4-2 cells than androgen-dependent LNCaP cells. These data suggested that FOXM1 might have an important role in AI PCa.

## RESULTS

### FOXM1 is highly expressed in PCa cells but faintly expressed in non-tumorigenic prostate epithelial cells

**Figure 1:**
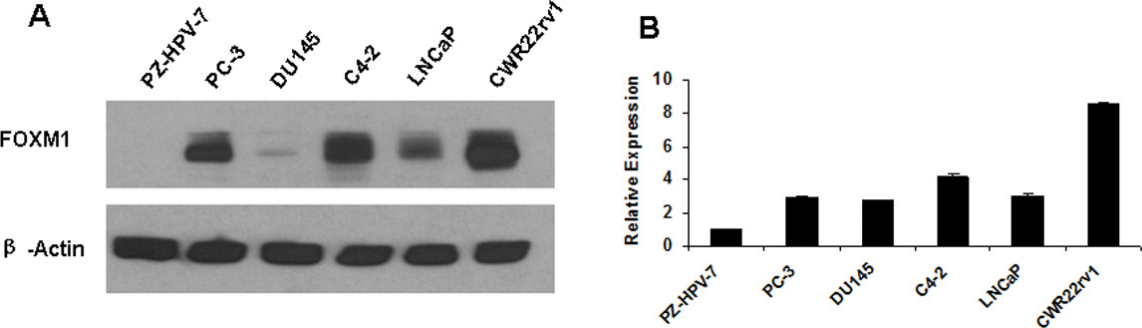
Gene expression of FOXM1 in human non-malignant prostate epithelial cells and prostate cancer cells (**A**) FOXM1 protein level was assessed in non-malignant PZ-HPV-7 prostate epithelial cells, androgen-dependent LNCaP cells, androgen-independent C4-2 and CWR22rv1 cells, and AR and PSA negative PC3 and DU145 cells by western blot. β-actin was used as the loading control. (**B**) FOXM1 mRNA was assessed by RT-PCR. FOXM1 was low in non-malignant PZ-HPV-7 prostate epithelial cells and higher in the tested PCa cell lines.

FOXM1 mRNA and protein expression are consistently at low levels in non-malignant PZ-HPV-7 prostate epithelial cells, while high levels of FOXM1 expression are detected in LNCaP, C4-2, CWR22rv1 and PC-3 PCa cells, only weak expression in DU145 cells. FOXM1 expression is higher in androgen independent C4-2 and CWR22rv1 cells than in androgen-dependent LNCaP cells (Figure 1A and 1B). Aberrant FOXM1 amplification may contribute to the development and progression of androgen-independent PCa.

### FOXM1 selectively regulated the transcription of androgen responsive genes

**Figure 2:**
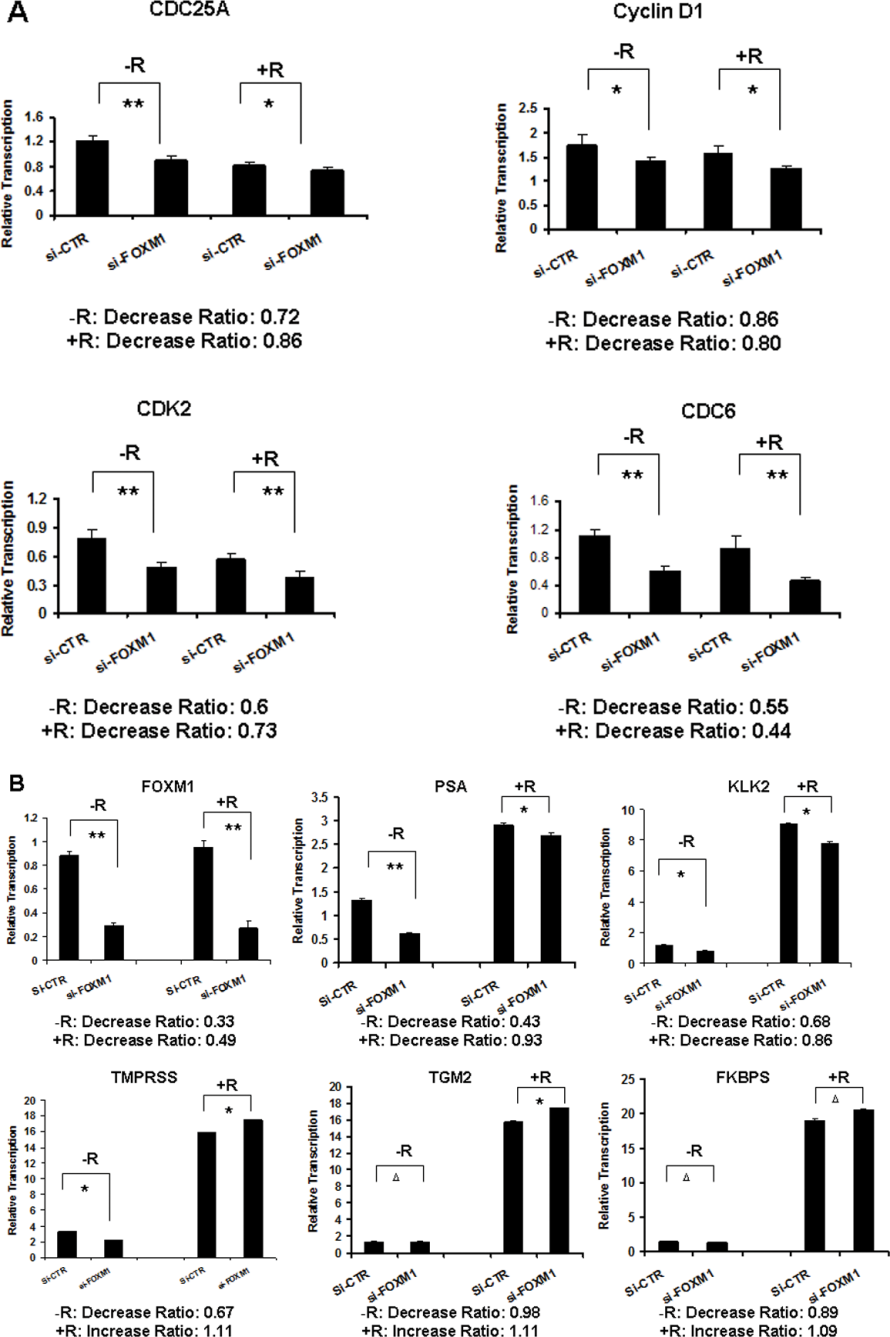
FOXM1 selectively regulated androgen-responsive gene transcription LNCaP cells were transfected with FOXM1 siRNA or non-specific control siRNA for 24 hours. The cells were treated with androgen (10 nM R1881) for an additional 16 hours. The mRNA levels of cell cycle regulatory genes and androgen responsive genes were tested by RT-PCR. *n* = 3. (**A**) Cell cycle regulatory genes (**P < 0.01, *P < 0.05). (**B**) Androgen-responsive genes (**P < 0.01, *P < 0.05, ^Δ^*P* > 0.05).

Since androgen/AR is critical in the development of PCa, we investigated the roles of FOXM1 in the transcription regulation of cell cycle regulatory genes and androgen responsive genes. We depleted FOXM1 in LNCaP cells using a siRNA, and tested the gene transcription of cell cycle regulatory genes CDC6, CDC25A, Cyclin D1 and CDK2, and androgen responsive genes PSA, KLK2, TGM2, TMPRSS2 and FKBP51 by RT-PCR. After FOXM1 depletion, the gene transcription levels of CDC6, CDC25A, Cyclin D1 and CDK2 decreased (Figure 2A). In the androgen responsive genes tested, only the mRNA levels of PSA and KLK2 decreased, while TMPRSS increased in the presence and absence of androgen with FOXM1 knockdown. FOXM1 knockdown did not decrease FKBP51 in the absence of androgen, but increased the mRNA level in the presence of androgen. TGM2 did not change in the presence or absence of androgen. The results indicated that FOXM1 selectively activated the transcription of androgen-responsive genes PSA and KLK2 (Figure 2B).

### Androgen did not affect FOXM1 expression, while FOXM1 increased AR gene expression and AR promoter activity

**Figure 3:**
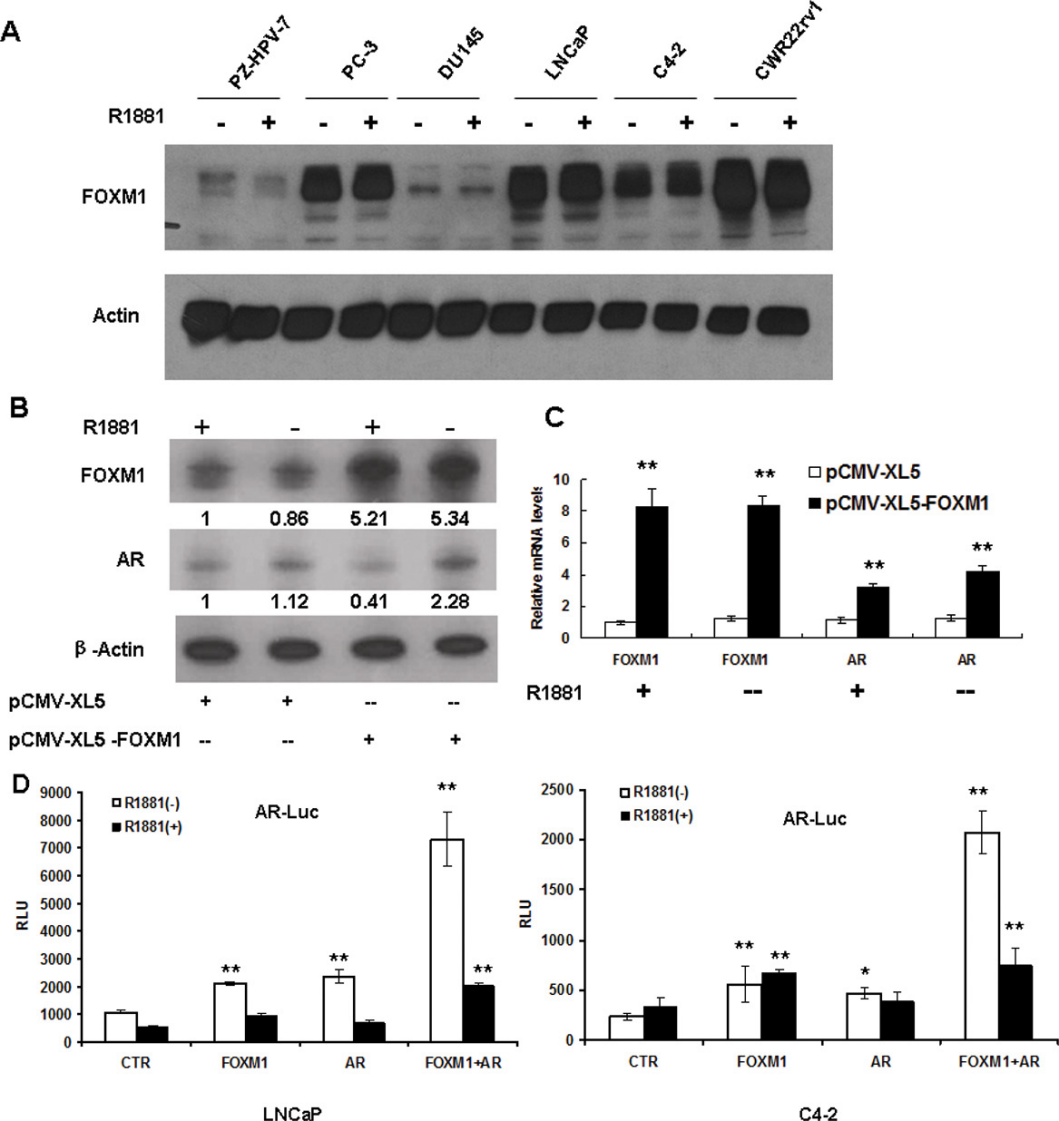
Androgen did not affect FOXM1 expression, while FOXM1 increased AR gene expression and AR promoter activity (**A**) Non-malignant PZ-HPV-7 prostate epithelial cells and prostate cancer cells were treated with or without R1881. FOXM1 protein was assessed by western blot, and β-actin was used as the loading control. (**B** and **C**) LNCaP cells were transfected with pCMV-XL5-FOXM1 or control vector for 48 hours, and cells were treated with R1881 for an additional 16 hours. FOXM1 and AR were tested at protein (B) and mRNA levels (C, **P < 0.01). (**D**) LNCaP and C4-2 cells were transfected with pGL3-AR-Luc, together with pCMV-XL5-FOXM1 (FOXM1), pCMV-XL5-AR (AR) or pCMV-XL5 (CTR), or FOXM1 plus AR together for 48 hours. The cells were treated with or without 10 nM R1881 for an additional 16 hours and assayed for luciferase activity. Results were expressed as mean+/− S.E. of triplicate reactions **P < 0.01, *P < 0.05).

Since FOXM1 was involved androgen-responsive gene transcription, we tested whether FOXM1 and AR activated mutually. We first tested FOXM1 protein expression in non-malignant prostate epithelial cells and PCa cells when the cells were treated with an artificially synthesized androgen R1881. No detectable FOXM1 response was found when cells were treated with R1881 (Figure 3A).

We then tested whether FOXM1 activated AR gene expression. LNCaP cells were transfected with FOXM1-expressing plasmids. Forty-eight hours post-transfection, the cells were treated with or without R1881 for an additional 16 hours. Protein expression was tested by western blot using antibodies against FOXM1 or AR. FOXM1 increased AR protein levels without androgen stimulation, while androgen slightly decreased AR protein levels (Figure 3B). We obtained similar results for mRNA levels. In the presence or absence of androgen, FOXM1 significantly increased the mRNA levels of AR (Figure 3C). To further clarify the mechanism by which FOXM1 elevated AR gene expression, we constructed an AR gene promoter and tested its activities when FOXM1 or AR was overexpressed in LNCaP cells and C4-2 cells. Without androgen stimulation, FOXM1 significantly increased AR promoter activity, and the combination of FOXM1 and AR further increased AR promoter activity. However, androgen did not further increase AR promoter activity in LNCaP and C4-2 cells (Figure 3D.). These results suggested that FOXM1 probably contributes to the progression of PCa through an AR pathway.

### FOXM1 alone and in combination with androgen/AR regulated PSA gene transcription

**Figure 4:**
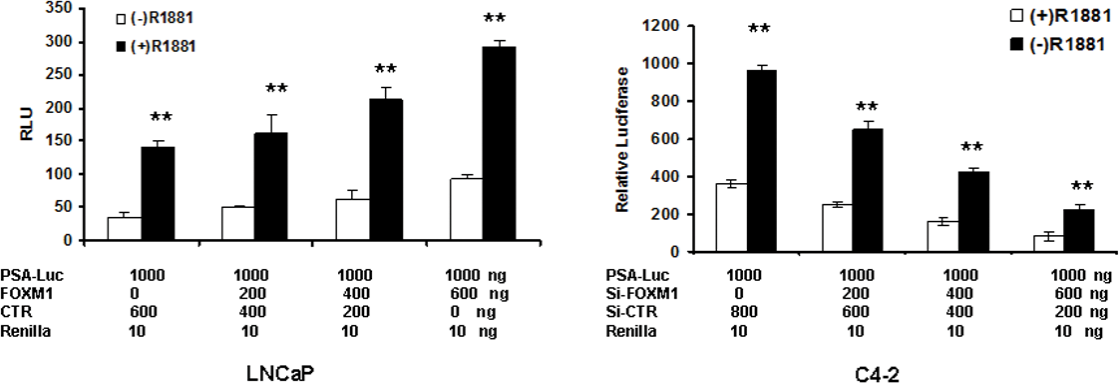
FOXM1 regulates PSA enhancer/promoter activity in the presence or absence of androgen LNCaP and C4-2 cells were co-transfected with PSA enhancer/promoter together with FOXM1 cDNA or FOXM1 siRNA at set doses for 48 hours. The cells were treated with 10 nM R1881 for an additional 16 hours. Luciferase activity was tested and renilla activity used as the transfection control. *n* = 4, **P < 0.01. (**A**) LNCaP cells. (**B**) C4-2 cells.

In low FOXM1-expressing LNCaP cells, FOXM1 increased the basal transcriptional activity of PSA promoter/enhancer in the absence of androgen. FOXM1 further increased PSA promoter/enhancer activity in the presence of androgen (Figure 4A). In high FOXM1-expressing C4-2 cells, the depletion of FOXM1 decreased PSA promoter/enhancer activity in the absence of androgen, and the depletion of FOXM1 further decreased androgen-increased PSA promoter/enhancer activity (Figure 4B). These data suggested that FOXM1, in addition to regulating AR gene transcription, probably regulates PSA gene transcription in an AR-independent manner.

### Several *cis*-regulatory elements of FOXM1 were identified in PSA promoter/enhancer regions, each immediately adjacent to an ARE

**Figure 5:**
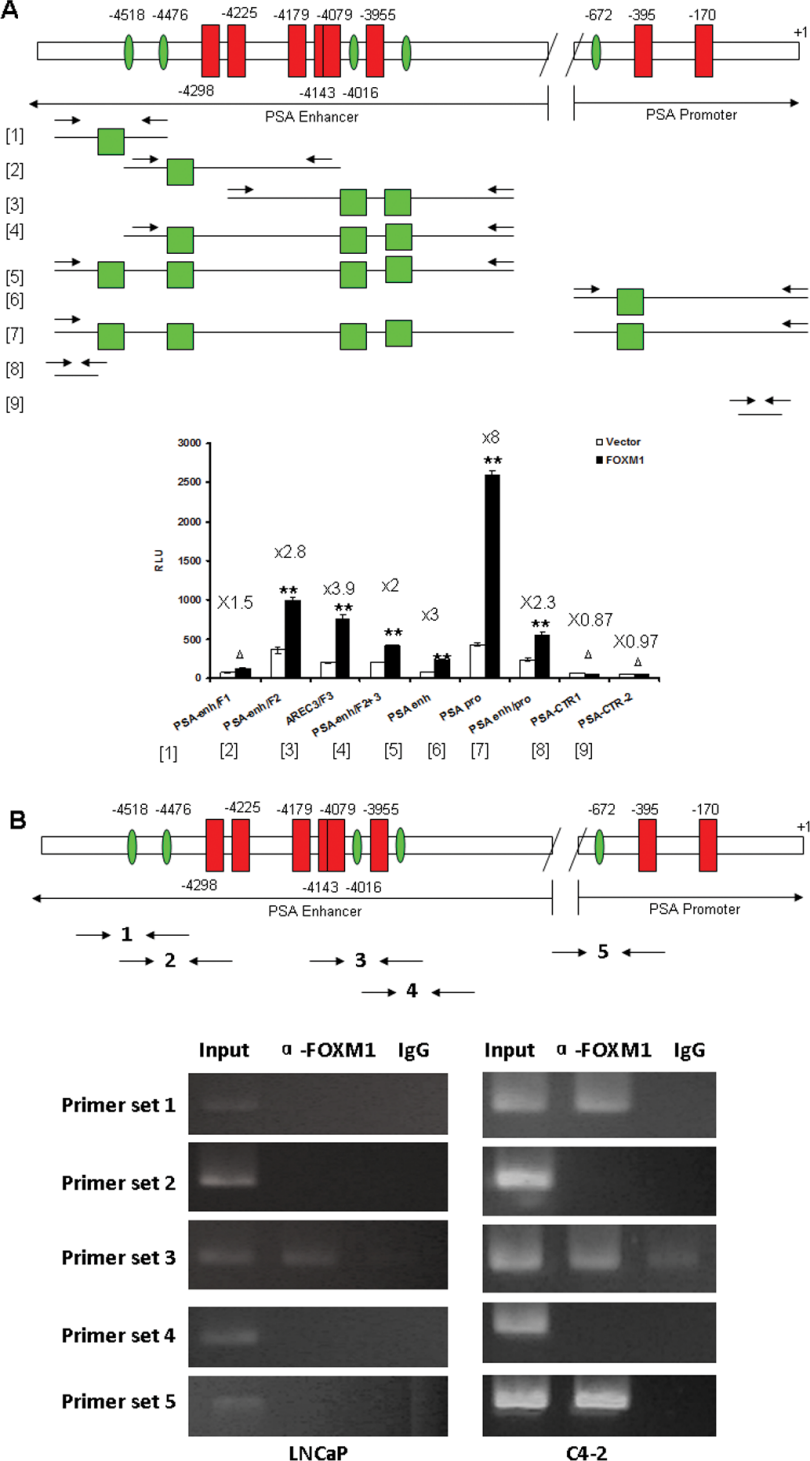
FOXM1 binding motifs were identified in PSA gene enhancer/promoter (**A**) Several promoter and enhancer fragments including or excluding the *cis*-regulatory element of FOXM1 were cloned, FOXM1 cDNA and PSA enhancer/promoter were co-transfected into LNCaP cells, and the transcriptional activity of PSA enhancer/promoter fragments was measured by dual luciferase assay. *n* = 4. (**B**) FOXM1 directly binds to PSA Promoter/Enhancer regions by ChIP-PCR. The chromatin DNA of LNCaP and C4-2 cells was immunoprecipitated (ChIP) by FOXM1 antibody. IgG was used as control. Five primer sets were designed for semi-quantitative PCR to test the direct binding of FOXM1 protein to PSA enhancer/promoter. (**P < 0.01, *P < 0.05, ^Δ^*P* > 0.05).

Screening potential DNA occupation of transcription factor-binding sites by the TESS program (http://www.cbil.upenn.edu/cgi-bin/tess/tess), we found 4 *cis*-regulatory elements of FOXM1 transcription factor (5′-A(C/T)AAA(C/T)AA-3′) within 5.3 kb to 3.7 kb of PSA enhancer (–3709 to –5292 upstream of transcription start site), and 1 *cis*-regulatory element within 677 bp (−672 to −660 upstream of transcription start site) of the PSA gene promoter. Each *cis*-regulatory element of FOXM1 is immediately adjacent to an individual ARE binding site. The adjacent localization of FOXM1 and AR binding sites in both PSA promoter and core enhancer suggested that FOXM1 and AR probably cooperate to regulate PSA gene transcription (Supplementay Figure 1). We cloned several promoter and enhancer fragments including or excluding the *cis*-regulatory element of FOXM1, and tested the impact of FOXM1 on the activities of these promoter/enhancer fragments. FOXM1 significantly increased the activities of PSA promoter/enhancer fragments containing FOXM1 binding sites but did not increase in fragments excluding FOXM1 binding sites (Figure 5A).

We tested whether FOXM1 regulated PSA enhancer/promoter activities by directly binding to the FHK binding motifs using ChIP-PCR. We designed five primer sets covering the individual FHK binding motifs for PCR after ChIP. Intriguingly, the binding status of FOXM1 was markedly different in androgen-dependent LNCaP and androgen-independent C4-2 cells. In LNCaP cells, the FOXM1 binding strength to the PSA enhancer/promoter was weak and only the FHK binding motif in the region of the AREC3 fragment of PSA enhancer was detected; no binding was detected for the other four FHK binding motifs. In C4-2 cells, in addition to the FHK binding motif in the region of the AREC3 fragment of PSA enhancer, FOXM1 binding was detected in FHK sites 1 and 5 respectively in PSA enhancer or promoter (Figure 5B). These results suggest a more important role for FOXM1 in androgen-independent PCa cells than androgen-dependent cells.

### Combination of AR and FOXM1 inhibitors further decreased PSA gene transcription

**Figure 6:**
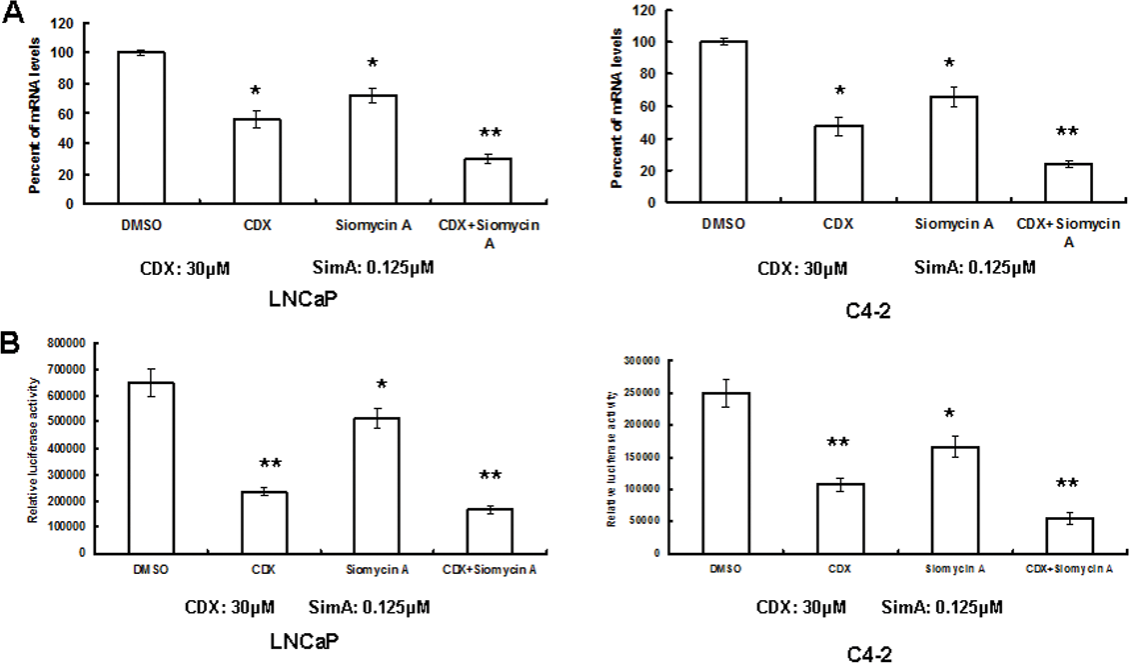
Combination of AR and FOXM1 inhibitors synergistically decreased PSA gene expression (**A**) LNCaP and C4-2 cells were treated with CDX or siomycin A alone or in combination for 24 hours. PSA mRNA was tested by RT-PCR. (**B**) The PSA enhancer/promoter was transfected to LNCaP and C4-2 cells, and then the cells were treated with CDX (30 μM) or/and siomycin A (0.125 μM) for 24 hours after DNA transfection. Luciferase activity was tested and renilla activity was used as the transfection control. *n* = 4, **P < 0.01, *P < 0.05.

Since the siRNAs of AR and FOXM1 alone decreased PSA gene transcription, we further tested whether the small molecular inhibitors of AR and FOXM1 can be used to inhibit PCa progression by that mechanism. In a previous study, we tested the combinational efficacy of FOXM1 inhibitor siomycin A and anti-AR agent Casodex (CDX) against LNCaP and C4-2 proliferation. Doses of siomycin A at 0.125 uM and CDX at 30 uM showed synergistic effects against cancer cell proliferation [16]. We tested whether siomycin A and CDX individually or in combination decreased PSA gene transcription at the same doses. As we expected, siomycin A and CDX alone decreased PSA gene transcription, and the combination of siomycin A and CDX further decreased PSA gene transcription (Figure 6A). We then found that siomycin A and CDX alone also decreased PSA enhancer/promoter activity, and the combination of siomycin A and CDX further decreased activity in both LNCaP and C4-2 cells (Figure 6B).

## DISCUSSION

In this study, we tested how PSA gene was regulated by FOXM1, a cell proliferation-specific transcription factor, in addition to androgen/AR in both androgen-dependent and androgen-independent PCa cells. FOXM1 was more highly expressed in PCa cells than in non-malignant prostate epithelial cells, suggesting that FOXM1 is a critical oncogene in PCa development. FOXM1 selectively regulated gene transcription of androgen responsive genes KLK2 and PSA in addition to cell cycle regulatory genes CDC25A, cyclin D1, CDC2 and CDC6. FOXM1 selectively binds to the FHK motifs in the genome, which is bound by other AR co-regulators such as FOXA1 in some cell lines [17]. The binding strength of FOXM1 to the promoter or enhancer region of androgen-regulatory genes is cell-dependent due to nuclear protein levels of FOXM1 and the chromatin status [18], which decide the selective regulation of target genes by FOXM1. In addition, FOXM1 regulates AR gene transcription, but was not regulated by androgen/AR, which is consistent to our previous studies [16]. We found that FOXM1 protein physically interacts with AR protein to form a transcription regulatory complex, and binds to the *cis*-regulatory consensus sequences of FOXM1 and ARE, which lie in close approximation at the CDC6 promoter [16].

**Figure 7:**
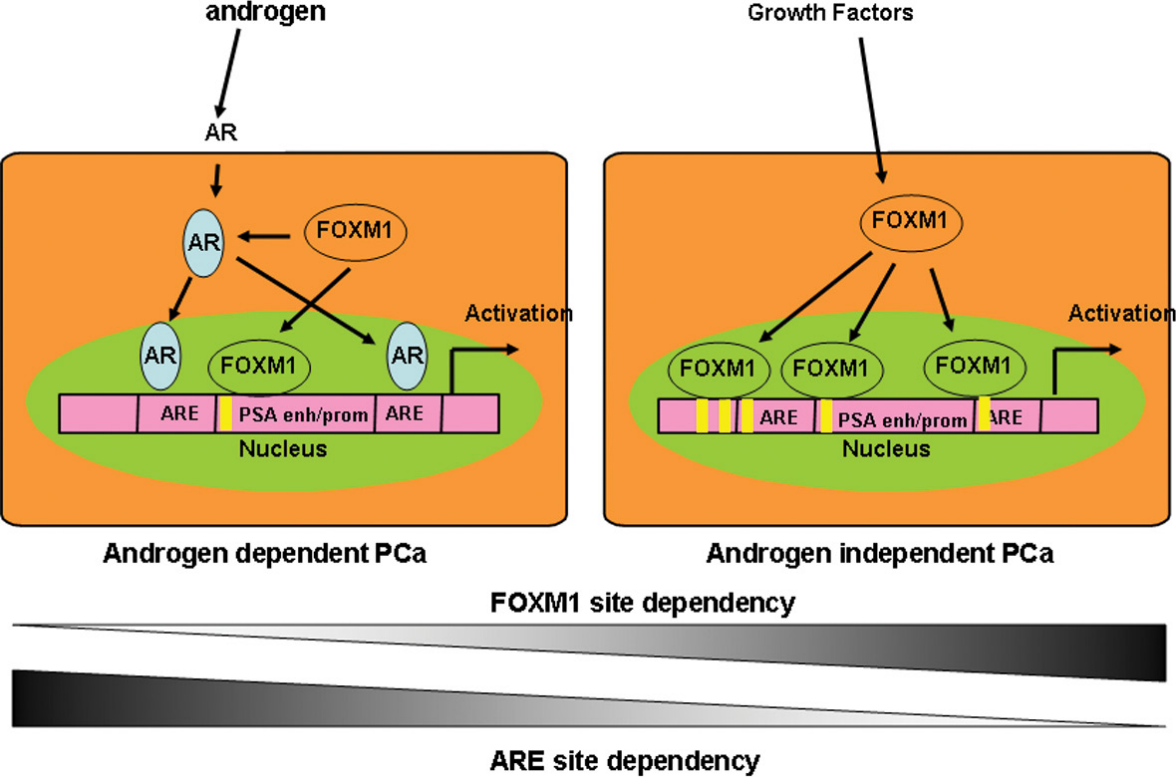
Schematic model of FOXM1 and AR cooperation in the transcriptional regulation of androgen-responsive genes in AD and AI PCa cells In androgen-dependent LNCaP cells, androgen/AR is more important than FOXM1 for regulating PSA gene transcription, while in androgen-independent C4-2 cells, FOXM1 takes over AR, and plays a more important role in PSA gene transcription.

The addition or depletion of FOXM1 markedly elevated or reduced PSA enhancer/promoter activity (Figure 4). In addition to regulating AR transcription, FOXM1 regulates PSA gene transcription in an AR-independent manner. Screening the potential DNA occupation of FOXM1-binding sites in the gene enhancer/promoter by ChIP-PCR, we identified four FOXM1 binding motifs in PSA promoter/enhancer regions. Intriguingly, the binding status of FOXM1 is different in androgen-dependent LNCaP and androgen-independent C4-2 PCa cells. More FOXM1 binding sites in the PSA enhancer/promoter were detected in C4-2 than LNCaP cells. These results suggested that while both AR and FOXM1 regulated PSA gene transcription, but the roles of FOXM1 are different in LNCaP and C4-2 cells. In androgen-dependent LNCaP cells, androgen/AR is more important than FOXM1 to regulate PSA gene transcription, while in androgen-independent C4-2 cells, FOXM1 takes over a more important role in PSA gene transcription (Figure 7). The depletion of AR and/or FOXM1 with siRNA or small molecular inhibitors alone or in combination decreased PSA gene transcription and PCa cell proliferation [16]. FOXM1 has great potential to as an important biomarker and as a therapeutic target [19]. Recent analysis of the genome-wide regulatory networks (interactomes) from expression profiles of human and mouse prostate cancer identified FOXM1 and CENPF together as robust prognostic indicators of poor survival and metastasis due to their synergistic regulation of signaling pathways associated with PCa malignancy [20, 21]. Our studies further validated FOXM1 as another therapeutic target in addition to AR to reduce PSA levels, particularly for androgen-independent PCa patients.

We explored the mechanisms by which FOXM1 and AR proteins interact to mutually regulate and co-activate PSA gene transcription. FOXM1 is higher in prostate cancer cells than the non-malignant prostate epithelial cells. FOXM1 selectively regulates the transcription of androgen/AR regulatory genes KLK2 and KLK3(PSA) in addition to the cell cycle-regulatory genes. FOXM1 binds to the FHK motifs in the promoter/enhancer of PSA gene, with more binding sites in androgen-independent C4-2 cells than androgen-dependent LNCaP cells. This study clarified novel mechanisms by which prostate cancer becomes androgen refractory, and identified FOXM1 as a potential biomarker and therapeutic target in advanced prostate cancer patients.

## MATERIALS AND METHODS

### Cell culture

PCa cell lines included AR-positive and androgen sensitive LNCaP (American Type Culture Collection, ATCC, Manassas, VA, USA), AR-positive and androgen insensitive C4-2 and CWR22rv1 (obtained from Dr. Chinghai Kao, Department of Urology, Indiana University School of Medicine) and AR-negative and androgen insensitive PC-3 and DU145 (ATCC). Cells were maintained in RPMI-1640 medium supplemented with 10% FBS and 1% penicillin/streptomycin. The non-malignant prostate epithelial cell line PZ-HPV-7 was purchased from ATCC, and the cells were cultured in keratunicyte serum free medium (K-SFM) with two additives, bovine pituitary extract and human recombinant epidermal growth factor (EGF).

### Plasmids, siRNA and shRNA

FOXM1 and AR cDNAs in the pCMV-XL5 vector were purchased from Origene (Rockville, MD, USA). pDRIVE01-PSAenh/prom(h)v04, containing a *PSA* gene enhancer (1582bp) and promoter (670 bp), was purchased from InvivoGen (San Diego, CA, USA). pGL3-AR-Luc was obtained from Dr. Chnghai Kao. pGL3-PSAenh/prom-Luc was generated by cutting the fragment of PSA enhancer/promoter out from pDRIVE01-PSAenh/prom(h)v04 with *Xba I* and *Nco I*. Then the PSA enhancer/promoter fragment was inserted to pGL3-basic vector. By screening the potential DNA occupation of transcription factor-binding sites by the TESS program (http://www.cbil.upenn.edu/cgi-bin/tess/tess), we found 4 *cis*-regulatory elements of FOXM1 transcription factor (5′-A(C/T)AAA(C/T)AA-3′) within 5.3 kb to 3.7 kb of PSA enhancer (−3709 to −5292 upstream of transcription start site), and 1 *cis*-regulatory element within 677 bp (−672 to −660 upstream of transcription start site) of the promoter of *PSA* gene. ON-Target plus SMARTpool small interfering RNA (siRNA) for the targets of AR and FOXM1 (target sequence CCAACAAUGCUAAUAUUCA), and the ON-Target plus non-targeting siRNA control were purchased from Dharmacon Inc. (Lafayette, CO, USA) via Thermo Fisher Scientific.

### Antibodies and chemicals

Specific antibodies including Anti-FOXM1 and anti-AR were purchased from Santa Cruz Biotechnology (Santa Cruz, CA, USA). The synergistic androgen methyltrienolone (R1881) was purchased from PerkinElmer (Walthan, MA). The antiandrogen compound Casodex and FOXM1 inhibitor Siomycin A were purchased from Sigma Aldrich (St. Louis, MO, USA).

### Transient transfection

LNCaP and C4-2 cells underwent DNA transfection using a Fugene 6 HD Transfection Kit (Promega, Madison, MI, USA). siRNA transfection was performed using DharmaFECT Transfection Reagent (Thermo Fisher Scientific, Wilmington, DE, USA). The experimental procedure slightly modified the protocol provided by the manufacturer.

### RT-PCR

Total RNA was extracted from PCa cells using the RNeasy Mini Kit (Qiagen, Valencia, CA, USA). The concentration of RNA was measured by a Nanpdrop 1000 Spectrophotometer (Thermo Fisher Scientific). One microgram of total RNA was used for reverse transcription to cDNA using the High Capacity RNA to cDNA Master Mix (Applied Biosystems, Carlsbad, CA). RT-PCR was run in the Applied Biosystems 7500 Real-Time PCR system. The primers specific for FOXM1, AR, PSA, KLK2, TMPRSS, TGM2, CDC6 and GAPDH were designed by Primer 3 software (version 0.4.0).

### Dual luciferase reporter assays

Cells were transfected with plasmids as described in the individual experiments, and plasmid pRL-SV40 (SV40 enhancer driven Renilla gene) was used as the transfection control. Cells were lysed 48 hours post-transfection with passive lysis buffer by dual luciferase assay kit (Promega). Luciferase activity was tested by a luminometer. The results were expressed as the ratio of the luciferase value to the Renilla value.

### ChIP-PCR

LNCaP or CWR22rv1 cells were transfected with or without siRNAs of AR, FOXM1 or non-targeting control, and the cells were harvested and used for ChIP assays. ChIP assays were performed using ChIP-IT Expression Chromatin Immunoprecipitation Kits (Active motif, Carlsbad, CA, USA). In brief, the cells were fixed with formaldehyde for 10 minutes, and the fixation reaction was quenched with Glycine Stop-Fix solution. The cells were lysed and sonicated until the desired lengths were achieved (100–500 bp). For the immunoprecipitation of formaldehyde cross-linked chromatin-protein complexes, the antibodies against FOXM1 were used or IgG was used as the negative control. The same amount of chromatin without antibody incubation was used as the input control. The samples were incubated on an end-to-end rotor for 3 hours at 4°C. The reaction mixtures were eluted following the manufacturer’s protocol. DNA was analyzed via semi-quantitative PCR. Primers spanning the FOXM1 binding sequence were designed, and primers spanning the regions lacking the FOXM1 binding sites were used as controls (for primer information see supplementary text). All ChIP experiments were repeated at least three times.

### Statistical analysis

The Microsoft Excel Program was used to calculate SD and statistically significant differences between samples using the Student’s *t* test. The asterisks in each figure indicate statistically significant changes with *P*s calculated by Student’s *t* test: *P < 0.05; **P ≤ 0.01 was considered statistically significant.

## SUPPLEMENTARY FIGURE


