# Supplementary material for: FOXM1 promotes the progression of prostate cancer by regulating PSA gene transcription
Source: Oncotarget. 2017 Feb 9;8(10):17027–37. doi: 10.18632/oncotarget.15224 (PMC5370019; doi:10.18632/oncotarget.15224)
Supplement: Supplementary file 1 [file oncotarget-08-17027-s001.pdf]

FOXM1 promotes the progression of prostate cancer by regulating PSA gene transcription

Supplementary Materials

Primers for FOXM1 binding sites in PSA enhancer

Primer set 1:

Forward: AGATAGAGTGCACAGGTCTGGA

Reverse: TTCCTCATGCTCCTGAAATAGA

Primer set 2:

Forward: GGTCCCCTCCTATCTCTATTCC

Reverse: GTAAAAGCCTTTGCCTCTGACT

Primers for FOXM1 binding sites in PSA promoter:

Primer set 5:

Forward: GCATGAGGATGATTCTGACCT

Reverse: GATCTGTGGACCACAAGATC

Primer set 3:

Forward: TCAGCCTTTGTCTCTGATGAAG

Reverse: CAGGCTTGCTTACTGTCCTAGA

Primer set 4:

Forward: GACAGTAAGCAAGCCTGGATCT

Reverse: TTTAATGGTGGAACGTTGAGAC

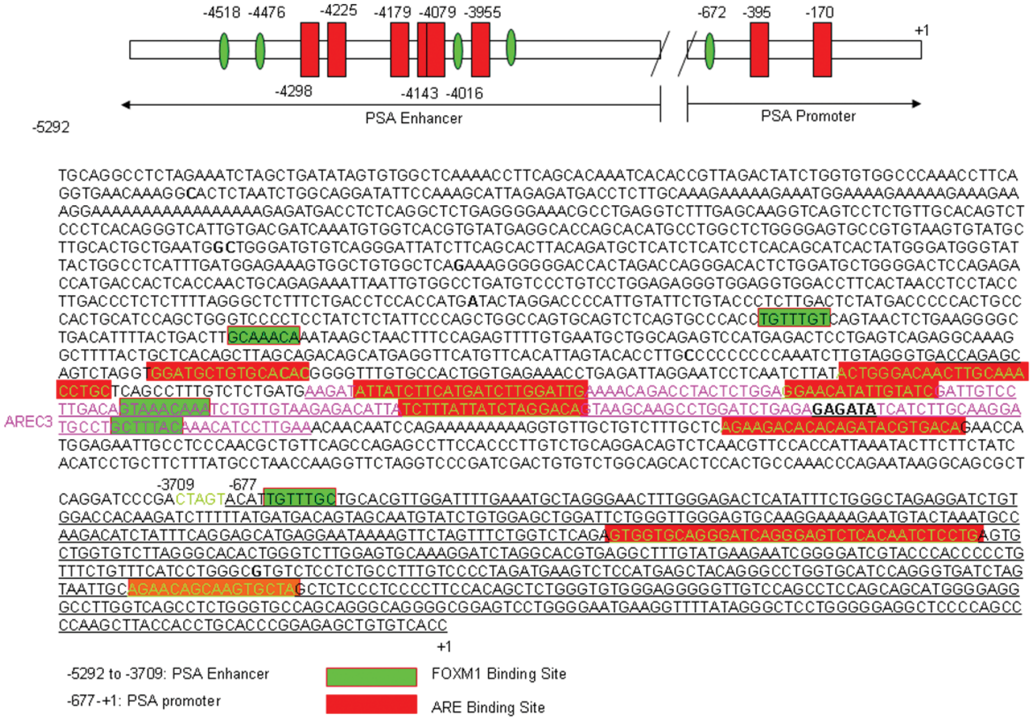

Supplementary Figure 1: Several FOXM1 cis-regulatory elements were identified in PSA promoter/enhancer regions, each immediately adjacent to an ARE. Schematic representation and gene sequence of PSA enhancer/promoter. The red boxes represent the androgen-responsive elements, and the green boxes represent the FOXM1 binding sites. Four *cis*-regulatory elements of FOXM1 have been identified within the PSA enhancer/promoter; three are located in the PSA enhancer regions, and one is located in the PSA promoter region. The putative FOXM1 binding sites are close to ARE binding sites.
